# A closed-loop audit of the prescription practice of topical antibiotics for acute infective conjunctivitis

**DOI:** 10.22336/rjo.2023.7

**Published:** 2023

**Authors:** Andreas Katsimpris, Nafsika Voulgari, Nikolaos Georgiadis, Stylianos Kandarakis

**Affiliations:** * First Ophthalmology Department, “G. Gennimatas” Hospital, Athens, Greece; National and Kapodistrian University of Athens, Athens, Greece; ** Department of Family and Community Medicine, School of Medicine, National and Kapodistrian University of Athens, Athens, Greece; “Ippokrateio” General Hospital of Athens, Athens, Greece

**Keywords:** antimicrobial resistance, primary care, audit, antibiotics, acute infective conjunctivitis

## Abstract

**Objective:** The implementation of guideline recommendations for antibiotics prescription for the management of patients with acute infective conjunctivitis (AIC) in primary care departments remains below par. Our objective was to assess the impact of clinical audit on adherence to evidence-based indications for prescription of antibiotic eye drops in patients diagnosed with AIC, in the setting of a primary care practice in western Greece.

**Methods:** We conducted a retrospective audit to evaluate the current prescription practice of antibiotics for the management of AIC. Following evidence-based indications for the prescription of antibiotics in AIC through literature search, and in combination with discussion and collaboration with the primary care doctors of our department, we formulated and implemented guidelines for the management of AIC. We then performed a prospective re-audit to assess the management of patients with AIC after local implementation of the guidelines.

**Results:** A total of 158 cases were audited in the first cycle before the introduction of the guidelines, from 15th June 2019 to 7th March 2020, and 26 cases after, from 10th March 2020 to 20th November 2020. The compliance with the guidelines regarding antibiotics prescription was significantly improved from 12.0% to 84.6% between the first and the second cycles of audit.

**Conclusions:** In this study, with the local introduction of guidelines, clinical audit significantly improved the prescription practice of topical antibiotics in patients with AIC in a primary care department.

**Abbreviations:** AIC = acute infective conjunctivitis

## Introduction

Acute infective conjunctivitis (AIC) is a common presentation in the primary care setting, comprising around 1% of primary care visits [**1**], with some of the most common symptoms of AIC being watery, sticky and/ or red eyes. It is difficult to distinguish viral and bacterial AIC based only on clinical examination, due to their similarities regarding ocular signs and symptoms of patients. Moreover, medical training is usually rudimentary when it comes to ophthalmology and, as a result, general practice doctors may lack the confidence and knowledge to properly manage patients with AIC or eye problems in general [**2**]. Despite, the high diagnostic accuracy of AIC in primary care practices [**3**], several studies have found an overuse of topical antibiotic treatment by primary care physicians [**4**,**5**], mainly due to the difficulty in the clinical differentiation between viral and bacterial AIC. 

AIC rarely causes loss of vision and the rate of resolution in untreated cases has been shown to be high in many randomized clinical trials [**6**-**8**]. Appropriate indications for prescribing antibiotics in patients with AIC exist [**9**] and should be followed by primary care practitioners to limit the needless use of antibiotics. Therefore, we performed an audit to assess the prescription practice of topical antibiotics for AIC in the setting of a primary care practice in western Greece. Moreover, we locally implemented a set of guidelines for the management of AIC and compared changes in practice before and after the introduction of the guidelines.

## Methods

This was a retrospective project and the population of the study represented all the patients in our primary care practice in Ano Chora, western Greece, discharged with a topical antibiotic prescription and the diagnosis of AIC from 15th June 2019 to 7th March 2020. The data regarding demographics, patient history and the details about topical antibiotic prescription were extracted from photocopies of the patients’ discharge notes and prescription data kept in the practice records. Microsoft excel was used to collect patient data.

After performing a literature search, we formulated the guidelines for prescribing antibiotics in patients diagnosed with AIC, based on the NICE guidance for AIC and on discussion with the five general practitioners in the department. The doctors easily identified the key features of the clinical assessment and diagnosing AIC, however, further discussion was needed for the treatment plan of AIC. It was agreed that antibiotics should be prescribed in 3 circumstances: 1) in patients with purulent discharge, 2) in contact lens wearers and 3) when conjunctivitis caused by chlamydia or gonorrhea was suspected. In all these cases, referral to ophthalmology was recommended. Additionally, all the doctors were informed about the indications for urgent referral of patients with conjunctivitis to ophthalmology as recommended by NICE [**10**]. 

The data was presented in the meeting attended by primary care physicians of the practice. The guidelines were introduced on 8th March 2020 and they were displayed in all the examination rooms of the department. Nurses and junior doctors actively encouraged the implementation of the guidelines and senior help was available if any clarifications needed to be offered regarding the management of patients with AIC. In addition, photocopies listing the indications regarding topical antibiotics use were handed to every physician and pinned to the board of doctors’ office. All physicians were also prompted to reevaluate any existing topical antibiotic use for AIC in every patient. After discussion with the clinical supervisor, the audit standard was set to an 80% of the prescriptions having a clear evidence-based indication. In early December, data from the discharge notes dated from 10th March 2020 to 20th November 2020 were extracted and the differences in the prescription practice of topical antibiotics for AIC before and after the introduction of the guidelines were assessed. 

*Statistical analysis*

Descriptive statistics of the study population were reported as percentage values for categorical variables and as mean ± standard deviation [median (min-max)] for continuous ones. Two-sided Student t-test or Mann-Whitney U test was used for continuous variables, and the Pearson chi-square test or Fisher exact test for categorical ones, to assess the differences [and 95% confidence intervals (95% CI)] between the variables of the study population before and after the implementation of the guidelines for the management of patients with AIC. All statistical tests were two-sided and p-values less than 0.05 were considered statistically significant, while all data analyses were conducted using R (version 3.5.2, Foundation for Statistical Computing, Vienna, Austria).

## Results

This audit covered 16 months, from June 2019 to November 2020. During this period, a total of 184 patients were identified (85 males and 99 females) in the records of the department, having been discharged with a topical antibiotic prescription and the diagnosis of AIC (**Table 1**). Regarding the demographics, the difference between the mean age of the patients before and after the implementation of the guidelines was not statistically significant [2.78 years (95% CI: -0.9 to 6.48 years)]. Similarly, sex did not statistically differ between the two groups.

**Table 1:** Characteristics of the patients diagnosed with AIC and treated with antibiotics (n = 184) before and after the introduction of the guidelines for the management of AIC

Parameters	Values		P-value
	Before 7th March 2020 N=158	After 10th March 2020 N=26	
Age (years)			
Mean ± SD	45.45 ± 8.24	48.23 ± 12.04	0.14 a
Median (min-max)	42.00 (21-83)	45.00 (20-79)	
Age group (years), n (%)			0.14 b
< 30	15 (9)	2 (8)	
30-39	43 (27)	3 (11)	
40-49	45 (28)	10 (39)	
50-59	33 (22)	4 (15)	
60-69	18 (11)	4 (15)	
> 70	4 (3)	3 (12)	
Sex, n (%)			0.90 c
Women	83 (53)	14 (54)	
Men	75 (47)	12 (46)	
Indication for antibiotic prescription, n (%)			< 0.01 b
No	139 (88)	4 (15)	
Yes	19 (12)	22 (85)	
AIC = acute infective conjunctivitis, SD = standard deviation, a Student’s t test, b Fisher exact test, c Pearson chi-square test			

From 15th June 2019 until 7th March 2020, a total of 158 patients (83 females and 75 males) were diagnosed with AIC and treated with antibiotic eye drops. However, only 19 (12%) of them had a clear indication for antibiotics prescription (**Table 1**). From 10th March 2020, when the guidelines for the management of patients with AIC were already implemented, until the end of the audit period, a total of 26 patients with AIC were prescribed topical antibiotics, of whom 22 (84.6%) had an indication for the prescription. There was a statistically significant difference, of 62.6% (Fisher’s exact test p-value < 0.001), regarding the percentage of proper prescription of topical antibiotics in patients diagnosed with AIC before and after the local introduction of the guidelines in our department. This difference revealed a marked improvement in compliance with guidelines regarding antibiotics prescription for treatment of AIC. The audit standard of 80% was met and all the general practitioners found the guidelines useful for the management of AIC. 

Finally, during the first cycle of audit, a total of 191 patients were diagnosed with AIC, of whom 158 (83%) received antibiotics (**Table 2**). These results are in accordance with the outcomes of other studies assessing the percentage of patients with AIC who were treated with antibiotics by non-ophthalmologists [**5**]. On the contrary, after the implementation of the guidelines, this percentage decreased to 19% (N=26), which is in line with the percentage of patients with AIC treated with antibiotics by ophthalmologists. 

**Table 2:** Total number of patients diagnosed with AIC and the reasons for being treated with topical antibiotics before and after the introduction of the guidelines for the management of AIC

Parameters	Values	
	Before 7th March 2020 N=158	After 10th March 2020 N=26
Patients diagnosed with AIC, n (%)	191 (100)	140 (100)
Patients diagnosed with AIC and treated without antibiotics, n (%)	33 (17)	114 (81)
Patients diagnosed with AIC and treated with antibiotics, n (%)	158 (83)	26 (19)
No clear indication	139 (73)	4 (3)
Purulent discharge	8 (4)	9 (6)
Contact lens wearer	8 (4)	11 (9)
Suspicion for chlamydia or gonorrhea	3 (2)	2 (1)
AIC = acute infective conjunctivitis		

## Discussion

This audit assessed the prescription practice of antibiotics in patients with AIC after the local implementation of guidelines during a 16-month period. Our closed-loop audit led to improvements in the pharmacologic treatment of AIC and increased the confidence and knowledge of general practitioners of our department on the management of AIC.

When discussing with the primary care physicians of the practice, the main reason identified for the non-etiological prescription of topical antibiotic treatment was the individual’s fear for possible complications when AIC was left untreated, even in the setting of clear evidence suggesting otherwise. Given that AIC severity can range from self-limiting infection to vision-threatening complications, such as endophthalmitis or keratitis, physicians in our department were afraid not to prescribe antibiotics. Moreover, the fastest resolution of symptoms in bacterial conjunctivitis treated with antibiotics compared to no-treatment [**11**], promoted this behavior. However, after considerate discussion and collaboration with the physicians, they were keen to change their prescription practice according to the guidelines that we introduced. The choice of antibiotics in our study was based on the findings from the clinical examination and history of the patient. In general, patients with mucopurulent discharge and contact lens wearers were treated with tobramycin ointment 3 times a day for 1 week, while patients with suspicion of gonococcal and chlamydial conjunctivitis were taken samples from conjunctival scrapings and/ or exudative fluid. If tested positive for gonorrhea and chlamydia, they were treated with intramuscular ceftriaxone 1 g and azithromycin 1 orally once, respectively.

AIC remains one of the most common ophthalmic conditions that physicians worldwide face in general practice, accounting for approximately 1% to 2% of total visits, comprising also a significant economic burden for many countries [**12**]. The type and prevalence of AIC depends on many factors, such as age of the patient, geographical location and seasonality. In general, viral conjunctivitis remains the most common cause of AIC in both the adult and overall population, followed by bacterial conjunctivitis, which is more common in children [**9**]. Apart from infectious causes of conjunctivitis, other types of conjunctivitis are toxic, cicatricial, allergic, as well as secondary to neoplastic and immune-mediated diseases, which further complicate the diagnosis and treatment plan of this entity. In addition, there are several sight-threatening conditions that can mimic AIC, so it is important for primary care doctors to be able to make proper decisions regarding referral, testing and treatment. Fortunately, several algorithmic approaches and guidelines exist for differentiating urgent eye conditions from AIC, which the primary care physician can easily adopt [**10**].

It has been shown that most patients with AIC are treated with antibiotics, although there are several factors influencing their treatment plan. First, patients with AIC are often prescribed antibiotics if they have visited non-ophthalmologists, such us general practitioners and pediatricians [**5**]. It has been shown that almost 70% of patients with AIC who have visited non-ophthalmologists were prescribed antibiotics, while this percentage significantly decreased to around 30% for patients who were treated by ophthalmologists [**5**]. Moreover, patients with lower socioeconomic status are less likely to get antibiotics prescribed for the treatment of AIC [**5**].

One of the most serious consequences of the excessive prescription of antibiotics is the increasing rates of antimicrobial resistance. Antimicrobial resistance imposes a significant challenge in many infectious diseases worldwide. Unfortunately, data regarding antimicrobial resistance of ophthalmic pathogens remain scarce not only in Greece, but also in several other countries [**13**]. Excessive clinical use and misuse of antibiotics for conditions other that AIC has been reported in many Greek hospitals and specifically in intensive care units [**14**]. The are many reasons for this condition; the Greek surveillance systems on antibiotics use lack effectiveness and reliability and they often inadequately disseminate knowledge and research information regarding antibiotics use. Additionally, due to the high number of patients attending the hospital departments in Greece, the relatively lack of shortage of doctors and the constant growth of the working hours, doctors may become overwhelmed and, as a result, there is often inadequate time for meaningful communication with the patient on antibiotics guidelines. 

## Conclusion

In conclusion, this primary care-based audit revealed that the implementation of local guidelines can significantly improve the prescription practice of antibiotics for the management of AIC in primary care departments. Although in our study the overall compliance regarding the appropriate use of antibiotics in AIC exceeded 80%, it was not perfect. Thus, the audit must be repeated after a period of time, allowing enough time for these recommendations to be established on long-term in current clinical practice.

**Conflict of Interest statement**

The authors state no conflict of interest.

**Informed Consent and Human and Animal Rights statement**

Informed consent has been obtained from all individuals included in this study.

**Authorization for the use of human subjects**

Ethical approval: The research related to human use complies with all the relevant national regulations, institutional policies, is in accordance with the tenets of the Helsinki Declaration, and has been approved by the review board of First Ophthalmology Department, “G. Gennimatas” Hospital, Athens, Greece.

**Acknowledgements**

None.

**Sources of Funding**

This research did not receive any specific grant from funding agencies in the public, commercial or not-for-profit sectors.

**Disclosures**

None.

**Contribution**

All the authors of the manuscript have equal contribution.
